# Validation of a Physical Activity and Health Questionnaire Evaluating Knowledge of WHO Recommendations among Colombians

**DOI:** 10.3390/ijerph18073526

**Published:** 2021-03-29

**Authors:** Sandra Milena Moreno-Lavaho, María Mendoza-Muñoz, José C. Adsuar, Jorge Carlos-Vivas, Jorge Rojo-Ramos, Fernando Manzano-Redondo, Jorge Pérez-Gómez

**Affiliations:** 1 Universidad del Tolima, Condominio La Samaria Casa C 15 Ibagué, Tolima 730006299, Colombia; smmorenolv@ut.edu.co; 2 Health, Economy, Motricity and Education Research Group (HEME), Faculty of Sport Sciences, University of Extremadura, 10003 Cáceres, Spain; mamendozam@unex.es (M.M.-M.); jadssal@unex.es (J.C.A.); jorge.carlosvivas@gmail.com (J.C.-V.); jorgerr@unex.es (J.R.-R.); jorgepg100@unex.es (J.P.-G.)

**Keywords:** knowledge, questionnaire, physical activity, World Health Organization, recommendations

## Abstract

Knowledge of physical activity (PA) can be considered a predictor of public health for society. Thus, this study aims to show content validity of the “Knowledge Questionnaire on World Health Organization (WHO) Recommendations on PA and Health” (CUAFYS-A) and reference values on adults’ knowledge of the WHO recommendations on PA. This is a quantitative, non-experimental, descriptive, and cross-sectional study, in which 579 adults completed an online questionnaire with demographic data. The questionnaire was made up of 9 items to measure PA related to knowledge. For the elaboration of the items of the questionnaire a disciplinary team formed it and for the analysis of results, a descriptive analysis of these was applied. Then an inferential analysis was performed, content validity, construct validity, and reliability were analyzed. The CUAFYS-A after its content analysis, obtained appropriate results in terms of pertinence and relevance; additionally, it showed Cronbach’s alpha coefficient of 0.62. Thereby, it was concluded the CUAFYS-A questionnaire proved to be a valid and reliable instrument to show reference values and to evaluate the knowledge of adults of PA and health according to the WHO recommendations.

## 1. Introduction

The World Health Organization (WHO) considers that lack of physical activity (PA) as the fourth most important factor determining the risk of mortality worldwide, constituting 6% of all deaths worldwide [1]. Therefore, PA is a very important factor that impacts people’s health. It is a personal task that arises from the self-discipline of caring for one’s own body and includes daily activities, such as household and work tasks, which involve some expenditure of energy, such as climbing stairs, moving from one place to another, playing a sport, etc. It has been proven that PA has many health benefits, such as reducing the risk of total mortality, the acquisition of cardiovascular disease, hypertension, obesity, etc. [2,3,4].

The world’s population is not performing the PA needed for health benefits. Therefore, the increase in sedentary lifestyles due to long working hours, as well as the use of electronic means for passive household and transport tasks, has led to a sharp decline in PA performance [1]. In this way, physically active individuals have a lower percentage of body fat than inactive people, of all ages and in both genders. However, as indicated by González-Gross and Meléndez [5], overweight individuals who are in good physical condition have better cardio-metabolic health than overweight, unfit individuals.

In order to clarify this issue, the WHO publishes in 2010 the global recommendations on PA for health [6], which have recently been expanded and update [7]. These recommendations are addressed to three different age groups (children and teenagers, adults, and older people), to prevent non-communicable diseases through the practice of PA in the whole population—such as cancer, diabetes, cardiovascular diseases, etc.

Thus, an important aspect of these recommendations will be to know the assessment of PA levels. Along these lines, there are different instruments for assessing the PA carried out by any group of society. These instruments must have as indispensable characteristics good validity and reliability so that they can be used for future research [8]. Likewise, all these instruments must undergo a validation process, which is a prerequisite for ensuring their quality for application [9]. Some authors, such as Galicia et al. [10], differentiate several types of validation methods when carrying out this process, such as construct validity, criterion validity, and content validity. Thereby, Haynes et al. [11] define content validity as “the degree to which elements of the measurement instrument are comprehensive, relevant, and representative of the construct for a particular assessment purpose” (p. 238). The most precise and purely objective ones used to measure PA, such as indirect calorimetry and direct observation, are hardly applicable and excessively expensive [12]. It announces that one of the most used techniques to estimate the level of PA in epidemiological studies is the PA questionnaire. Since it is a practical and simple tool to determine the level of PA in older people, this type of instrument is very appropriate to study the level of PA in large populations [13]. The ease of administration, the minimal burden on respondents, and the low cost were highlighted among other factors [14].

As can be observed in several studies, there are many PA questionnaires such as The International PA Questionnaires (IPAQ) developed by Zanchetta et al. [15], which measures health-related PA in different age groups. The PA questionnaire for adolescents [16], which measures the level of PA. The Global PA Questionnaire (GPAQ V2) [17], which measures PA levels (active rest, moderate and vigorous) and sedentary time. The Netherlands PA Questionnaire (NPAQ), developed by Bielemann et al. [18], measures the PA preferences of children aged 4 to 11 according to parents in Brazil. Finally, the Girls health Enrichment Multi-site Studies (GEMS) Activity Questionnaire (GAQ) [19], which serves to measure the level of PA and sedentary time, in a group of people from a university community in Colombia.

However, all these studies focus on measuring PA practice itself or the level of PA that its participants have. Otherwise, from a knowledge perspective of the relationship between PA and disease, an online survey of 615 people was also conducted to measure the current level of PA, as well as their level of knowledge of the benefits and risks of PA [20]. Results showed that participants were significantly more active when they identified more diseases associated with physical inactivity. Studies such as Fredriksson [20] show the importance of increasing knowledge of the types of diseases associated with inactivity, as the low level of this knowledge suggests that it should be promoted more. This will guide the frequency, types, and duration of physical examination and activity required for health [20]. Another study, such as Hui et al. [21], aims to assess PA-related knowledge in adults in China about how exercise influences well-being. In this study, a 20-item questionnaire was developed, and the level of PA was measured by the International PA questionnaire. In conclusion, a positive association with the level of PA was obtained. The level of education significantly influenced the association between knowledge and level of PA, leading to the suggestion of vulnerable groups to aim for improvement in PA versus risking [21].

Therefore, it should be noted that health programs aim to promote the acquisition of specific knowledge about PA, to help people manage and cope with health conditions such as sedentary lifestyle, obesity, and hypertension. In this way, people’s health can be better assessed through the use of consistent tools to evaluate knowledge of PA recommendations suggested by the WHO [22]. These tools, which allow the assessment of specific knowledge, are important as they can help people identify those individuals who specifically need intervention for sedentary lifestyles. They can also help to assess the effectiveness of PA programs as suggested by the WHO [22].

In this way, it is clear that there are several types of questionnaires related to PA, but, to our best knowledge, there are virtually none that are reliable and that measure knowledge of adults of the WHO recommendations on PA and health.

According to Ayona et al. [23], there is insufficient knowledge among adults and older people about the advice, importance, potential, and preventive nature of PA. All this is added to the fact that on many occasions, there is little disposition of this population towards the practice of physical activities, conditioned by little motivation, ignorance, and lack of free time.

Therefore, this study aims to show content validity of the “Knowledge Questionnaire on WHO Recommendations on PA and Health” (CUAFYS-A) and reference values on adults’ knowledge of the WHO recommendations on PA.

## 2. Materials and Methods

### 2.1. Participants

The sample consisted of 579 people, 280 men (48.4%) and 299 women (51.6%) between the ages of 18 and 65. These participants were selected according to the following inclusion criteria: (a) Colombian nationality; (b) female and male gender; (c) acceptance of participation through informed consent; (d) no disability, either hearing or visual; (e) not having a degree in PA and Sport Sciences; and (f) complete 100% of the questionnaire.

This study was approved by the Bioethics Committee of the University of Extremadura (66/2020) and was abides by the Declaration of Helsinki of the World Medical Association [24] which promotes the dignity of persons engaged in health research and the protection of their well-being. Furthermore, the participants before answering the questionnaire (“Knowledge Questionnaire on WHO Recommendations on PA and Health”, CUAFYS-A) read and signed the informed consent. All participants were selected in the municipality of Ibagué-Tolima.

### 2.2. Methods and Instrument

**Figure 1:**
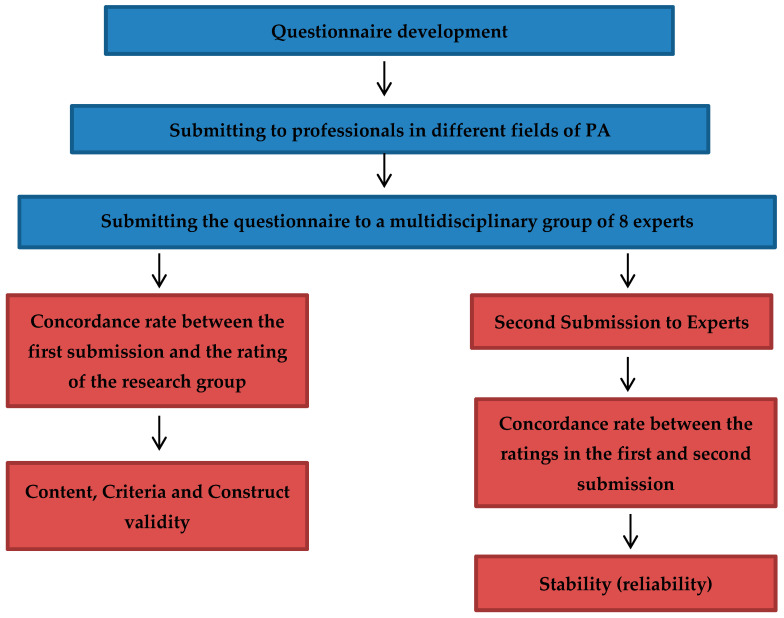
Development and validation of a questionnaire on knowledge of WHO PA Recommendations.

The research was of minimal risk, according to the categories stipulated by Resolution 8430 of 1993 of the Colombian Ministry of Health [25]. This study was quantitative, non-experimental research, with a descriptive and transversal scope, where the CUAFYS-A (Table A1) was developed and validated. This was applied to determine the knowledge that adults have about the WHO recommendations. According to Morera-Castro et al. [26] or Grau [27], the specific procedures for the construction of an instrument are literature review, instrument design or development of the questionnaire, submitting the questionnaire to a multidisciplinary group of experts, validation of the questionnaire (validity and reliability), and data processing and statistical analysis (Figure 1).

Following a brief review of the literature, then, the questionnaire was developed, consisting of partially open and closed questions on issues considered important, thus involving the following areas of PA included in the WHO global recommendations (concepts, recommendations, recommended levels for PA and population health, exercise, and activities of daily living). A disciplinary team made up of professionals in different fields of PA participated in the design of the CUAFYS-A questionnaire. The moderator of the group was a professional in PA and sport science, who led the discussions, which were written by an observer (researcher). Thus, this questionnaire aimed to generate current questions on PA recommendations according to the WHO [6].

In the subsequent stage, the questions were analyzed by eight expert graduates in PA and sport science. Each question and the alternative were analyzed according to whether the item was: essential, useful but not essential, and not necessary, following these categories designed by Lawshe [28], to be used by experts in the content evaluation of each item. Only questions that had received scores above 0.58 were included in the questionnaire, following Lawshe’s model [28] modified by Tristán [29,30], where it is stressed that the content validity ratio must be equal to or greater than 0.58 to be accepted. The eight experts identified the following problems when analyzing the questionnaire: in initial questions 1 and 12, the content was repeated; question 6 was opinion-based so it did not correspond to the objective of the questionnaire; for question 8, the wording was modified to Likert type. For this reason, it was decided to remove these four questions. Also, the questionnaire was answered by 40 young people and adults (from 18 to 65 years old) with different academic levels. No problems of linguistic comprehension were detected in any of the items presented by the participants in the pilot. Finally, the questionnaire was composed of nine questions and three possible answers (I agree, I disagree, and I don’t know).

The validation stage checked the quality of the questions, i.e., whether the CUAFYS-A questionnaire assessed the knowledge of the WHO recommendations on PA. To verify the content validity, it was analyzed whether the questionnaire covered all relevant topics on the WHO PA recommendations and whether the questions fit each item, made based on the judgment of the disciplinary team members, previously made.

To verify the construct validity, the questionnaire was applied to 40 young people and adults (from 18 to 65 years old) with different academic levels as indicated above.

The internal reliability of the instrument was calculated using Cronbach’s alpha coefficient, which defined the accuracy and stability of the questionnaire.

### 2.3. Statistical Analysis

A descriptive analysis was first performed with the IBM-SPSS 26 statistical package (Chicago, IL, USA) and the descriptive statistics of the sample were calculated, with medians and standard deviations and with a level of a priori statistical significance lower than 0.05.

First, normality and homogeneity were tested using the Kolmogorov–Smirnov test and Levene’s test, respectively. As outcomes revealed that data did not follow a normal distribution (*p* > 0.05), the Mann–Whitney U test was applied to analyze between-sex differences. Then, a descriptive and inferential statistical analysis of the results was performed where content validity was calculated through expert judgment where the content validity ratio (CVR) of content relevance was obtained. This quantitative index is Lawshe’s CVR [28], later modified by Tristan’s CVR’ [29,30]. Concerning construct validity, the number of factors present in the questionnaire was examined, and in turn, the Kaiser–Meyer–Olkin sample adequacy index and the Bartlett sphericity test were evaluated [31]. The internal consistency of the questions was evaluated using Cronbach’s alpha coefficient [32]. Finally, three categories are established to present the cut-off values of the questionnaire. The first threshold corresponds to poor knowledge, being those participants who have less than 50% correct answers to the questionnaire; the second threshold corresponds to sufficient knowledge, for participants with a percentage of correct answers between 50–75%; and the third threshold corresponds to good knowledge, for participants with a percentage of correct answers higher than 75%.

## 3. Results

Demographic questions on age, gender, height, and weight, and these results are shown in Table 1. The variable “Do you consider yourself to be physically active?” was also introduced, with the possibility of answering yes or no, as well as the variable relating to the education of the participants, analyzing four different levels according to the Colombian Ministry of Education: High School, technical level (relating to professional technical programs), technological level (relating to technological programs), and professional level (relating to professional programs). These three levels of training correspond to the undergraduate level.

Taking into account the results obtained from the sample (Table 1), it shows that women percentage of female participants outweighed 51.6% compared to the percentage of men 48.4%, for a total of 579 participants. The median age of women was 23.0 years old, for men 21.0 years old and the total median for both sexes was 22 years old. Men had a significantly higher body weight (*p* < 0.001) 70 kg than women 60 kg, and men were significantly taller (*p* < 0.001) 173 cm than women 160 cm. Concerning the median weight and height, it can show that women present a BMI of 23.5 kg/m^2^, while the BMI for men is 23.7 kg/m^2^.

The median age between both sexes was 22 years and more than 50% were considered physically active, being 56.9% of women and 81.4% of men. Related to this, for the variable “If considered physically active”, it was observed that the majority of people of the total (68.7%) responded that they were considered active and a smaller percentage (31.3%) considered themselves physically inactive. Education of participants was analyzed at four different levels according to the Colombian Ministry of Education showed results in ascending order: 37.5% had a high school level, 17.1% a technical level, 11.4% a technological level, and 34% a professional level.

As for content validity, after obtaining the observations of the specialists consulted, corrections were made to the questions that presented a CVR’ index of less than 0.58 (CVR and CVR’), following the procedure of Lawshe [28]. It is important to note that the questions themselves had no observations from the people consulted and had a CVR’ of 0.85.

The Content validity was carried out by a group of experts, who evaluated it individually and according to their experience according to the criteria of authors such as Tristán-López or Vargas Salgado [29,30] (Table 2).

Referring now to the validity of the construct, as it is a new instrument, the number of factors present in the questionnaire was examined. The Kaiser–Meyer–Olkin sample adequacy index (KMO = 0.74) and the Bartlett sphericity test (*p* < 0.001) were evaluated. The questionnaire was also found to be valid.

Finally, regarding reliability, internal consistency and stability of the questionnaire were established by Cronbach’s alpha, resulting in a coefficient α = 0.62 (*n* = 579) which was interpreted as acceptable for scientific purposes, being between 0.6 and 0.7 according to Nunnally and Bernstein [32].

Thus, Table 3 shows the total knowledge score according to sex differences (*n* = 579). A total score of 67.31 is obtained, with men scoring slightly higher than women.

In addition, the values obtained for adults’ knowledge of the WHO recommendations on PA are shown in this Table 4. This table shows how in questions 1, 7, and 8, many of the participants fail in their answers, while in the rest of the questions, most of them get the right answers. Considering the level thresholds set as cut-off values of the questionnaire, it shows that 71 participants have poor knowledge (12.3%), 317 have sufficient knowledge (54.8%), and 191 have good knowledge (32.9%).

## 4. Discussion

The main aim of this study is to show content validity of the CUAFYS-A questionnaire and reference values on adults’ knowledge of the WHO recommendations on PA.

The questionnaire is an increasingly relevant part of the evaluation of scientific evidence, as there are no other known methods or studies on assessing PA knowledge and WHO recommendations. Therefore, PA researchers should know which statistical procedures allow them to optimize the measurement of their variables of interest, and through the empirical results of this study, content validity is demonstrated, which is necessary for this purpose.

The results in Table 1, show that women present a BMI of 23.5 kg/m^2^, while the BMI for men is 23.7 kg/m^2^, which indicates that both sexes are ‘normal weight’, according to the WHO classification [33] and recommendations [6]. Men show a slightly higher level of high school education than women, and show very similar values at the technical and technological levels, in line with the study of Romero and Urbina [34]. It also shows that 81.4% of men consider themselves physically active, compared to 56.9% of women. This may be because women tend to do less PA than men. This is consistent with the study by Casado and del Villar Álvarez [35], who show that women do 16% less PA than men and that 46.7% of women aged between 18 and 25 consider themselves physically inactive, which could be a key factor to be taken into account in future WHO recommendations or actions.

According to the Lawshe Model [28] modified by Tristán-López [29], the content validity of the instrument of 0.85 has been proved, indicating that it presents acceptable psychometric properties to be used as an evaluation instrument, as a minimum of 0.58 is accepted to validate the item. After finding the CVR’ for each item, and following the criteria posed by the Lawshe Model [28], it is obtained that the questionnaire is made up of 9 items. In Table 2, all items have a CVR index’ above 0.75, showing that they comply with the acceptable value [29]. The results of the validation by expert judgment indicate that a CVR = 0.69 was obtained for each question of the questionnaire. The validity index of the items considered acceptable is 0.85, so it is possible to say that they are acceptable. However, this allows us to point out that from the perspective of academics who are experts in the field; some aspects are relevant to the study. Thus, after the evaluation made by the experts, the questionnaire is considered valid. In this way, similar results are observed in the design study of the Childhood PA Pictorial Questionnaire (C-PAFI) [26]. The results of the validation by expert judgment in its research indicate that a CVR = 0.99 was obtained for each question of the questionnaire. A positive and favorable CVR was found for the variables in most of its dimensions and indicators, achieving a CVR of 81% for the whole test, which is very satisfactory as most of the instruments obtained a score above 0.58, considered the minimum allowed.

Otherwise, for the methods derived from the application of the instrument itself, the analysis of main components presents a clear orientation towards the concordance and correlation between the items themselves, with which they are understood as methods closer to the study of construct validity. The Kaiser-Meyer-Olkin sample adequacy index was 0.74, being above 0.6, which is the limit marked as poor. Likewise, Barlett’s test of sphericity tests was used to check that the matrix is an identity matrix. In this test, if the significance is greater than 0.05, it cannot be assured that the factor model is adequate. In this case, *p* < 0.001, so it is considered valid.

This analysis makes it possible to identify the explanatory level of the factors that make up the variable studied and subsequently validate the items. Despite this, it is not possible to conclude that—having found factors such as the knowledge of how much PA, and the risks and benefits of PA—the variable is multidimensional.

Positive results are obtained in the reliability of the final questionnaire resulting after the judging with nine items, as well as by variables, whose global internal consistency through Cronbach’s alpha coefficient is 0.62, which is considered to be of acceptable consistency, showing a value between 0.6 and 0.7, according to the scale followed by Nunnally and Bernstein [32].

Thereby, continuing with the analysis of the internal consistency can be considered adequate, since Cronbach’s Alpha as a method used for the reliability calculation is high and allows confirming that the answers are related to each other. This makes it possible to conclude that all items measure the variable as a whole. Therefore, they can be added up into a total score to measure each factor and to the scale integrally.

Table 3 shows the total score obtained from all participants following their responses to the CUAFYS-A questionnaire. Ramírez-Clavijo [36] shows the importance to be aware of these recommendations in PA, simply to be able to develop them, for example with one’s children, grandchildren, nephews, etc. Knowing how to transmit the importance of these recommendations in PA will promote the healthy growth and development of healthy habits in children and any person in general, as well as guaranteeing good health for the rest of their lives. Also, this Table 3 shows that there are significant differences (*p* = 0.023) between men and women after performing the Mann–Whitney U test on the total knowledge score. Nevertheless, a correct total score of 67.31 is achieved.

Then, Table 4 shows the predominant values extracted from the participants’ answers to the questionnaire. In this way, there is some agreement with the aforementioned study by Ayona et al. [23], which states that there is not enough knowledge about all the aspects surrounding PA in the adult population, which may lead to many people not doing the recommended amount of PA simply due to a lack of knowledge. In this line, the value thresholds established in the questionnaire show that most of the participants in the study have sufficient knowledge (54.8%), followed by good knowledge (32.9%).

Among the strengths of the study, it is possible to identify the homogeneity of the sample in terms of socio-demographic characteristics and the heterogeneity in terms of the areas of knowledge to which it belongs. Furthermore, even though a slight increase in knowledge is observed in people who are related to the area of PA and health, in general, the deficiencies expressed by people are similar. Another of the strengths of this study was that the questionnaire is a simple tool to evaluate the knowledge that adults have about the WHO recommendations on PA and health.

The main limitation of this study is that we are based on the PA recommendations agreed by the WHO in 2010 [6], being aware that these recommendations have recently been updated [7] and as the study was carried out before this update. However, to the best of our knowledge, these recommendations have simply been expanded and may be used for future research with the questionnaire applied here or for an update. Also, the limitations of the study include non-probability sampling for convenience. This leads to the conclusions of the study being considered as previous hypotheses for further research, in which probability sampling is carried out and knowledge is examined knowledge about the above mentioned PA recommendations using this questionnaire in a wider and different population than that studied here. Another limitation is that being a self-reporting measure, the results are subjective according to each individual’s perception, which may overestimate or underestimate their knowledge of the PA recommendations indicated. It is also pointed out that test–retest was not performed and is possible that the result of the questionnaire was acquired knowledge.

## 5. Conclusions

In summary, the CUAFYS-A questionnaire is designed to evaluate adults’ knowledge of PA and health according to recommended by the WHO in 2010. A nine-item questionnaire has been developed and a descriptive analysis of the 579 participants was carried out. In this way, the results show a content validity of the instrument of 0.85, which indicates that it has acceptable psychometric properties to be used as an evaluation instrument. The tests carried out have been allowed us to identify the explanatory level of the factors and made it possible to validate the different items. Also, Cronbach’s Alpha coefficient shows a value of 0.62, revealing acceptable internal consistency, stability, and accuracy.

This study shows the percentage of correct answers of the participants to each question of the questionnaire, as well as the total score of knowledge of these WHO recommendations on PA, with a score of 67.31, finding significant differences according to the sex. Only 12.3% of the participants have a poor knowledge below the established value thresholds, most of them obtaining sufficient knowledge.

It is shown that it is important to know the procedure to be able to carry out a study on the validation of the content of an instrument such as this questionnaire. We believe it is advisable to use the CUAFYS-A questionnaire, as it is feasible and quickly administered, it has acceptable characteristics, and has been validated both by experts and by content validation, showing acceptable measures of internal consistency and validity. Finally, it can be stated that the questionnaire has a considerable potential to elucidate the degree of knowledge that adults have of PA and health recommendations according to WHO.

## Figures and Tables

**Table 1:** Characterization of the sample.

Variables		Women *n* = 299 (51.6%)	Men *n* = 280 (48.4%)	Total *n* = 579	*p*
Age (years old)	Median (IR)	23.0 (15)	21.0 (8)	22 (11)	<0.001 *
Weight (kg)	Median (IR)	60 (14)	70 (17)	65 (16)	<0.001 *
Height (cm)	Median (IR)	160 (8)	173 (7)	167 (13)	<0.001 *
Body Mass Index (BMI)	Median (IR)	23.5 kg/m^2^	23.7 kg/m^2^	23.6 kg/m^2^	<0.001 *
“Do you consider yourself to be physically active”	Yes (*n*/%)	170 (56.9)	228 (81.4)	398 (68.7)	<0.001 **
	No (*n*/%)	129 (43.1)	52 (18.6)	181 (31.3)	
High School	*n* (%)	107 (35.8)	110 (39.3)	217 (37.5)	<0.001 *
Technical Level	*n* (%)	50 (16.7)	49 (17.5)	99 (17.1)	
Technological Level	*n* (%)	34 (11.4)	32 (11.4)	66 (11.4)	
Professional Level	*n* (%)	108 (36.1)	89 (31.8)	197 (34)	

* Mann–Whitney U test for sex comparison; ** X^2^ test; IR = interquartile range.

**Table 2:** Content validity.

Questions	Essential	Useful But Not Essential	Not Necessary	CVR Lawshe	CVR’ Tristán
1. Children aged 5 to 17 should do vigorous activities at a maximum 3 times a week (playing football, basketball, swimming…).	7	1		0.75	0.88
2. Activities that involve bone stress, such as running, jumping rope or lifting weights.	7	1		0.75	0.88
3. For children aged 5 to 17 years, doing more than 1 h of PA every day (such as brisk walking, cycling, swimming…) can be detrimental to their health.	7	1		0.75	0.88
4. According to the WHO in children and young people, PA is considered to be: playing, sports, travel, recreational activities, physical education or scheduled exercise (with the family, at school or in everyday life).	6	1		0.50	0.76
5. For children aged 5 to 17 years, doing more than one hour a day of PA (such as brisk walking, cycling) can be beneficial to their health.	7	1		0.75	0.88
6. Children aged 5 to 17 years should do at least 1 h of PA every day? (such as walking, cycling, swimming…).	7	1		0.75	0.88
7. According to the WHO in children and young people, PA is considered to be: doing sports, physical education or programmed exercise (with the family, at school or in their daily life).	6	1		0.50	0.76
8. Children from 5 to 17 years old should do every day a maximum of 1 h of PA (such as fast walking, cycling…).	7	1		0.75	0.88
9. Children aged 5 to 17 years should do vigorous activities at least 3 times a week (playing football, basketball, swimming).	7	1		0.75	0.88
				6.25	7.68
				0.69	0.85

CVR represents the degree of consensus of the expert panel (*n* = 8) regarding whether or not the reagent is essential to achieve the objective of the questionnaire. CVR Lawshe = Content Validity Ratio of Lawshe; CVR’ Tristan = Content Validity Ratio of Tristán. CVR index must be equal to or higher than 0.58 to be accepted, following the Lawshe and Tristan models.

**Table 3:** Total score and sex comparison in CUAFYS-A.

		Women *n* = 299	Men *n* = 280	Total *n* = 579	*p* *
Total Score	Media (SD)	65.87 (13.89)	68.85 (12.90)	67.31 (13.50)	0.023

* Mann–Whitney U test for sex comparison.

**Table 4:** Percentages obtained according to participants’ responses (*n* = 579).

Questions	I Agree *n* (%)	I Disagree *n* (%)	I Don’t Know *n* (%)
1. Children aged 5 to 17 should do vigorous activities at a maximum 3 times a week (playing football, basketball, swimming…).	424 (73.2%)	**133 (23.0%)**	22 (3.8%)
2. Activities that involve bone stress, such as running, jumping rope or lifting weights.	**461 (79.6%)**	96 (16.6%)	22 (3.8%)
3. For children aged 5 to 17 years, doing more than 1 h of PA every day (such as brisk walking, cycling, swimming…) can be detrimental to their health.	164 (28.3%)	**400 (69.1%)**	15 (2.6%)
4. According to the WHO in children and young people, PA is considered to be: playing, sports, travel, recreational activities, physical education or scheduled exercise (with the family, at school or in everyday life).	**519 (89.6%)**	45 (7.8%)	15 (2.6%)
5. For children aged 5 to 17 years, doing more than one hour a day of PA (such as brisk walking, cycling) can be beneficial to their health.	**492 (85.0%)**	71 (12.3%)	16 (2.8%)
6. Children aged 5 to 17 years should do at least 1 h of PA every day? (such as walking, cycling, swimming…).	**487 (84.1%)**	76 (13.1%)	16 (2.8%)
7. According to the WHO in children and young people, PA is considered to be: doing sports, physical education or programmed exercise (with the family, at school or in their daily life).	**262 (45.3%)**	301 (52.0%)	16 (2.8%)
8. Children from 5 to 17 years old should do a maximum of 1 h of PA every day (such as fast walking, cycling…).	385 (66.5%)	**177 (30.6%)**	17 (2.9%)
9. Children aged 5 to 17 years should do vigorous activities at least 3 times a week (playing football, basketball, swimming).	**479 (82.7%)**	81 (14.0%)	19 (3.3%)

The correct option is shown in bold.

## Data Availability

The datasets used during the current study are available from the corresponding author on reasonable request.

## Appendix A

**Table A1:** Original Questionnaire.

CUESTIONARIO CUAFYS ADULTOS
1. Los niños de 5 a 17 años deben hacer actividades vigorosas como MÁXIMO * 3 veces a la semana (Jugar a fútbol, a baloncesto, nadar...). *Considerándose vigorosa aquellas que conlleven un esfuerzo □Totalmente de acuerdo □En desacuerdo □No sé
2. Los niños de 5 a 17 años, Deben realizar actividades en las que se produzca ESFUERZO ÓSEO por ejemplo, correr, saltar, nadar o levantar pesas. *Entendiéndose, como actividad física la que tiene por objeto incrementar la fortaleza en determinados puntos de los huesos del aparato locomotor. □Totalmente de acuerdo □En desacuerdo □No sé
3. En niños de 5 a 17 años, hacer más de 1 hora de actividad física TODOS LOS DIAS (como andar rápido, montar bici, nadar...) puede ser perjudicial para su salud. □Si □No □No sé
4. Según la OMS en niños y jóvenes, considera que hacer actividad física es: jugar, hacer deportes, desplazamientos, actividades recreativas, educación física o ejercicios programados (con la familia, en la escuela o en su día a día). □Totalmente de acuerdo □En desacuerdo □No sé
5. En niños de 5 a 17 años, hacer más de una hora al día de actividad física (como andar rápido, montar bici, nadar) puede ser beneficioso para su salud □Muy beneficioso □Nada beneficioso □No sé
6. Los niños de 5 a 17 años, deben realizar TODOS los días como MÍNIMO 1 hora al día de actividad física (como andar rápido, montar en bici, nadar ...) □Totalmente de acuerdo □En desacuerdo □No sé
7. Según la OMS en niños y jóvenes, se considera que hacer actividad física es SOLO: hacer deportes, educación física o ejercicios programados (con la familia, en la escuela o en su día a día). □Totalmente de acuerdo □En desacuerdo □No sé
8. Los niños de 5 a 17 años, deben realizar TODOS los días como MÁXIMO 1 hora al día de actividad física (andar rápido, montar en bici, nadar ...). □Totalmente de acuerdo □En desacuerdo □No sé
9. Los niños de 5 a 17 años deben hacer actividades vigorosas como MINIMO 3 veces a la semana (jugar a futbol, baloncesto, nadar) Considerándose vigorosamente aquellas que conlleven un esfuerzo de 7–8 sobre 10. en una escala donde 0 a 10. □Totalmente de acuerdo □En desacuerdo □No sé
